# Lactic Acid Bacteria Isolated from Japanese Fermented Fish (Funa-Sushi) Inhibit Mesangial Proliferative Glomerulonephritis by Alcohol Intake with Stress

**DOI:** 10.1155/2018/6491907

**Published:** 2018-02-11

**Authors:** Yumiko Yamada, Masumi Endou, Shunichi Morikawa, Jun Shima, Noriko Komatshzaki

**Affiliations:** ^1^Nodakamada Gakuen, 389-1 Noda, Noda, Chiba 278-0037, Japan; ^2^Department of Human Nutrition, Seitoku University, 550 Iwase, Matsudo, Chiba 271-8555, Japan; ^3^Department of Anatomy and Developmental Biology, Tokyo Women's Medical University, 8-1 Kawada-Cho, Shinjuku, Tokyo 162-8666, Japan; ^4^Faculty of Agriculture, Ryukoku University, 1-5 Yokotani, Seta Oe-cho, Otsu, Shiga 520-2194, Japan

## Abstract

The aim of this study was to examine the effect of heat-killed *Lactobacillus paracasei* NFRI 7415 on kidney and bone in mice fed an ethanol-containing diet with stress. Eight-week-old Cril  :  CD1 mice were fed a control diet (CD), an alcohol diet (AD) (35.8% of total energy from ethanol), or an alcohol diet containing 20% heat-killed *Lb. paracasei* NFRI 7415 (10^7^ CFU/g) (LD) for 4 weeks. Mice in the AD and LD groups also underwent restraint stress for two weeks from 13 days. The mice were placed in a 50 mL plastic tube, which had a small hole drilled around its base to allow ventilation, and restrained for 1 h every day. High final body weight was in the following order: CD, LD, and AD (*p* < 0.05). The heat-killed *Lb. paracasei* NFRI 7415 lowered liver total cholesterol concentration and plasma glutamic-oxaloacetic transaminase (GOT) level. In addition, fecal bile acids of the LD group were higher than in the AD group (*p* < 0.05). The glomerulus of the kidney in the AD group was observed to be more fibrotic than in the CD and LD groups with azan stain. Immunostaining confirmed that brown areas indicating the existence of mesangial cells were increased in the AD group, but not in the CD and LD groups. These results indicated that the heat-killed *Lb. paracasei* NFRI 7415 inhibited mesangial proliferative glomerulonephritis by alcohol intake with stress.

## 1. Introduction

People are subjected to many stressors in modern life, including both physical and mental stresses. Excessive stress can cause psychosomatic disorders, and sometimes death from overwork [1]. In recent years, lifestyle-related diseases are in most cases caused by overwork, excessive stress, sleep deprivation, smoking, and drinking [2]. It is well known that chronic ethanol consumption induces osteoporosis and chronic nephritis [3–5].

Lactic acid bacteria (LAB) have been utilized as a natural health food since ancient times, and the health-promoting effects of LAB are well recognized [6]. Some LAB are used in food fermentation, and typical examples can be found in the dairy industry for the production of cheese, yogurt, and other fermented milk products [7, 8].

Recent studies have indicated that several LAB are effective as probiotics for the prevention of osteoporosis and chronic nephritis. For example, *Bifidobacterium longum* alleviated bone loss in ovariectomized rats and enhanced bone mass density [9]. It was reported that yogurt consumption retarded chronic kidney disease progression [10].

*Lactobacillus paracasei* NFRI 7415, isolated from traditional Japanese fermented fish (funa-sushi), showed high *γ*-aminobutyric acid (GABA)-producing ability [11]. We reported that *Lb. paracasei* NFRI 7415 removed cholesterol from the plasma and liver of rats fed an ethanol-containing diet [12]. Moreover, it was shown that this strain reduced the content of liver lipids in C57BL/6J mice fed a high-fat diet [13]. We speculated that *Lb. paracasei* NFRI 7415 might have improved liver function in the abovementioned clinical study by somehow reducing hepatic lipid content. To the best of our knowledge, no study has investigated the effect of *Lb. paracasei* in the kidney of rat with stress and chronic ethanol consumption.

The aim of the present study was to examine the effect of heat-killed *Lb. paracasei* NFRI 7415 on the kidney and bone in mice fed an ethanol-containing diet with stress. We investigated body and fat tissue weight, as well as calcium in the bone and tissues of the kidney in mice. Symptoms associated with glomerulonephritis are hyperlipidemia and proteinuria [14]. It has been reported that supply of amino acid-fortified low-protein diets to nephritic rats improved their symptoms, and fecal bile acid excretion was enhanced [15]. Thus, we focused on the effect of fecal bile acid excretion by *Lb. paracasei* NFRI 7415 and investigated the fecal lipids of mice.

## 2. Materials and Methods

### 2.1. Preparation of Extract

A preculture of *Lb. paracasei* NFRI 7415 was grown to the stationary phase at 37°C for 20 h in MRS agar medium (Difco Laboratories, Detroit, MI). The medium was prepared by mixing an alcohol diet (AD) and sterilized water at a ratio of 1  :  3. The precultures (10^7^ CFU/g) were inoculated in 0.4 liters of AD at 37°C for 48 h. The medium was then heated at 100°C for 30 min and used in the animal experiments.

### 2.2. Animals and Diets

Twenty-four 8-week-old Cril  :  CD1 mice were purchased from Charles River Japan (Yokohama, Japan). All animals were housed individually in plastic cages in a controlled environment of 22 ± 1°C at 50% relative humidity under a 12 h dark/light cycle (19:00–7:00). The animals were randomly divided into three dietary treatment groups with equal mean body weight: the control diet (CD) group (*n* = 8), the alcohol diet (AD) group (*n* = 8), and the heated medium with *Lb. paracasei* NFRI 7415 (10^7^ CFU/g) and alcohol diet bended at a ratio of 1  :  4 (LD) group (*n* = 8).

**Figure 1:**
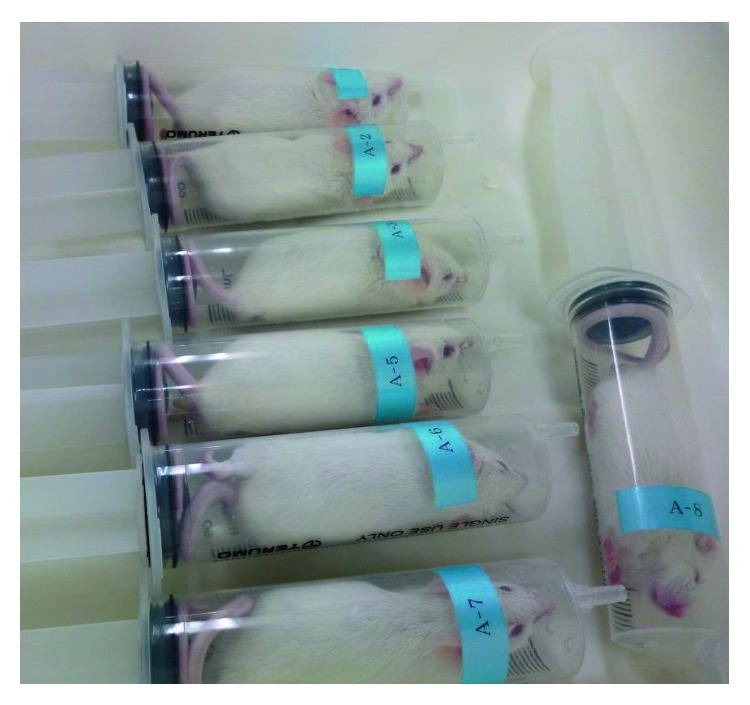
Restraint stress. Mice were placed in a 50 ml plastic tube, which had a small hole drilled around its base to allow ventilation, and restrained for 1 h every day.

Composition of the liquid diets (CD, AD, and LD) is given in Table 1; diets were formulated with reference to the Lieber–DeCarli diet [16]. All liquid diets were freshly prepared on alternate days. The mice were fed the CD, AD, or LD for 4 weeks. Food intake was recorded daily, and body weight was measured on alternate days. The mice of AD and LD group were subjected to restraint stress for two weeks from day 13 after feeding. Restraint stress was made with reference to the method of De Francesco et al. [17]. The mice were placed in a 50 mL plastic tube, which had a small hole drilled around its base to allow ventilation, and restrained for 1 h every day (Figure 1). Then, experimental mice were returned to their home cages. After the feeding period, the mice were fasted for 16 h and sacrificed humanely under ether anesthesia to collect the liver, kidney, and perirenal fat tissue. The blood was collected by heart puncture with a heparinized syringe. The blood was maintained at 4°C and centrifuged at 1,000 g for 15 min; the plasma and liver were stored at −80°C until analysis. All procedures were performed in accordance with the Animal Experimentation Guidelines of the Laboratory Animal Care Committee of Seitoku University (approval number 179).

### 2.3. Biochemical Assays of Plasma and Liver

Liver lipids were extracted by the method described in the work of Folch et al. [18]. Triacylglycerol (TG), total cholesterol (T-cho), HDL-cholesterol, glutamic-oxaloacetic transaminase (GOT), and glutamic-pyruvic transaminase (GPT) in plasma and liver extracts were measured using test kits (Triacylglycerol E-Test, Cholesterol E-Test, HDL-cholesterol E-Test, and Transaminase CII-Test, Wako Pure Chemical Industries, Osaka, Japan).

### 2.4. Assays of Ash, Calcium, and Phosphorus

The right femur was taken and weighed after removing the meat. Ash content in the femur was first treated by drying at 105°C for 48 h, then defatted by absolute ether for 3 days. Defatted bone was treated by ashing process at 550°C overnight in an electric furnace. The ash in the femur was measured with the weight method [19]. Ash was then added to 10 mL of 2 N HCl, and calcium and phosphorus in ash were measured with test kits (Calcium E-Test and Phospha C-Test, Wako Pure Chemical Industries, Osaka, Japan).

### 2.5. Assay of Fecal Lipids and Fecal Cholesterol

Here, 0.1 g of homogenized dry fecal matter was added to 4 mL of concentrated sulfuric acid in test tubes for 30 min at room temperature. Diethyl ether was added to reach 25 mL, and mixed. The diethyl ether-containing layer was moved to a flask, and the diethyl ether was evaporated. The fecal lipid in the flask was then weighed. The T-cho concentration in fecal matter was determined in the same way as in the liver T-cho concentration.

Fecal bile acids were measured by the procedure described in the work of Iwami et al. [20]; 10 mg of the sample was mixed with 0.2 mL of 90% ethanol during vortex mixing and incubated for 1 h at 65°C. The mixture was subjected to centrifugation at 5,000 rpm for 3 min. The supernatant was transferred to a 1.5 mL tube, and the ethanol was evaporated. Then, 0.2 mL of 90% ethanol was added in the precipitate for vortex mixing. The sample was dissolved in 1 mL of 90% ethanol and measured using test kits (total bile acid test by enzyme colorimetric method, Wako pure Chemical Industries, Osaka, Japan).

### 2.6. Kidney Histology

Kidneys of mice from the three dietary treatment groups were compared histologically. Under deep anesthesia with ether, the chest of a mouse from the three groups was opened rapidly and the vasculature was perfused with 50 mL of a fixative (4% paraformaldehyde in 0.01 M sodium phosphate-buffered saline (PBS: pH 7.4)) at a pressure of 120 mmHg from a 18-gauge cannula inserted into the aorta via an incision in the left ventricle. Immediately after fixative perfusion, the kidney was removed, cut into small pieces, and immersed in the same fixative overnight at 4°C. Kidney pieces were then washed with PBS, dehydrated in an ascending series of ethanol aqueous solutions (70%, 80%, 90%, and 100%), cleared in xylene, and embedded in paraffin wax. Three-micrometer-thick sections of paraffin-embedded kidneys were then subjected to hematoxylin and eosin (H&E) staining by a routine procedure (Meyer's hematoxylin staining, followed by eosin Y staining) and azan staining by a routine procedure (Mordant, Mallory's azocarmine G solution, 5% phosphotungstic acid solution, and Mallory's aniline blue orange G stain solution). Samples were then examined under a microscope (Olympus CH20 with Shimazu Moticam 580).

### 2.7. Immunostaining

Deparaffinized and rehydrated 3 *μ*m paraffin sections of formaline-fixed kidneys were enzyme-treated by protease. The primary antibody used for this study was desmin (West Grove, PA, USA). Secondary antibodies used peroxidase-conjugated AffiniPure Donkey Anti-Rabbit IgG (H + L), purchased from Jackson ImmunoResearch (West Grove, PA, USA). Liquid DAB + Substrate Chromogen System (Dako, Tokyo, Japan) were used for the color reaction, after counterstaining with hematoxylin. For confirmation of the mesangial proliferative glomerulonephritis by inducing ethanol intake and restraint stress, the number of the mesangial cells was counted in the 65 glomerulus of the kidney.

### 2.8. Statistical Analysis

Values were expressed as mean ±SD. Repeated-measures analysis of variance (ANOVA) was used to evaluate the effects of groups. Differences in mean values between groups were tested by Scheffe multiple-range test. A *p* value of less than 0.05 was considered statistically significant.

## 3. Results

### 3.1. Food Intake, Total Energy Intake, Body Weight, Liver, Kidney, and Perirenal Fat Tissue Weight

**Figure 2:**
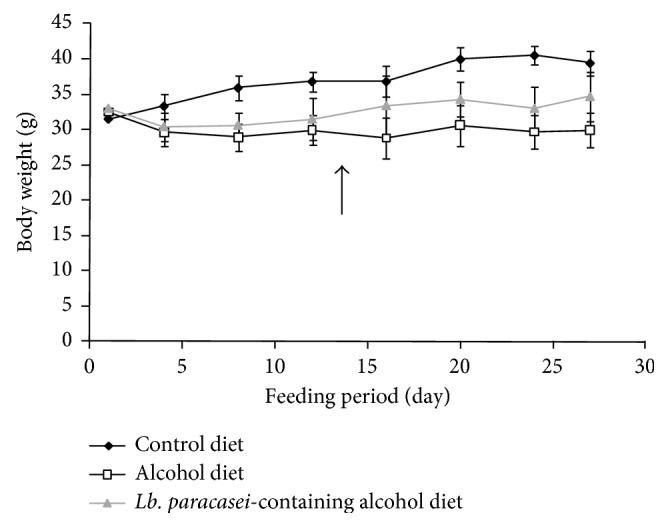
Body weight of mice during the experiment (*n* = 8). Arrow: start of restraint stress.

No significant differences in liver and kidney weights were observed among the three groups. Total energy intake and perirenal fat tissue weight of the AD and LD groups were lower than in the CD group (*p* < 0.05) (Table 2).  Figure 2 shows the body weight of mice during the experiment. The final body weight of mice was high in the following order: CD, LD, and AD (*p* < 0.05). After the mice in the AD and LD groups underwent restraint stress from 13 days, weight between the AD and LD groups increased.

### 3.2. Plasma Lipids Profiles and Liver Lipids

**Figure 3:**
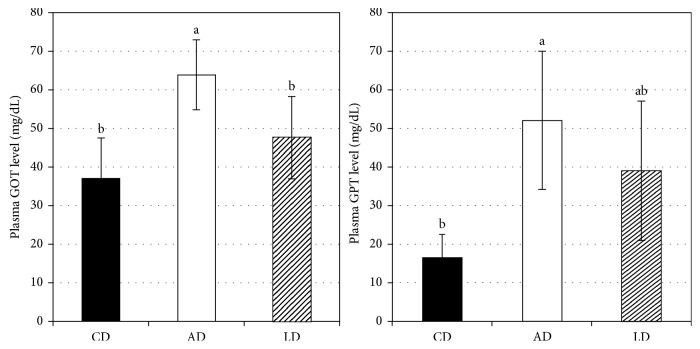
Plasma GOT level and plasma GPT level concentration of mice fed experimental diets. CD, control diet; AD, alcohol diet; LD, *Lb. paracasei*-containing alcohol diet. Values represent mean ±SD; *n* = 8. Values not sharing a common superscript letter are significantly different at *p* < 0.05.

Although no significant differences in plasma TG concentration were observed between the three groups, the plasma T-cho and HDL-cholesterol concentrations of the AD group were lower than those of the CD and LD groups (*p* < 0.05) (Table 3). No differences were observed in liver total lipids and liver TG concentrations between groups, but liver T-cho concentrations of the CD and LD groups were lower than those of the AD group (*p* < 0.05) (Table 3). The plasma GOT level of the AD group was higher than those of the CD and LD groups (*p* < 0.05) (Figure 3). In addition, the plasma GPT level of the AD group was higher than that of the CD group (*p* < 0.05) (Figure 3).

### 3.3. Bone Contents, Plasma Calcium, and Fecal Lipids

The ash content in the bone of the AD group was lower than those in the CD and LD groups, although there were no significant differences in right femur weight, calcium, or phosphorus in the bone between the groups (*p* < 0.05) (Table 4). There was also no difference in plasma calcium between groups. No significant differences were observed in fecal T-cho concentrations, but the fecal bile acids of the AD group were lower than those of the CD and LD groups (*p* < 0.05) (Table 4).

### 3.4. Kidney Histology

**Figure 4:**
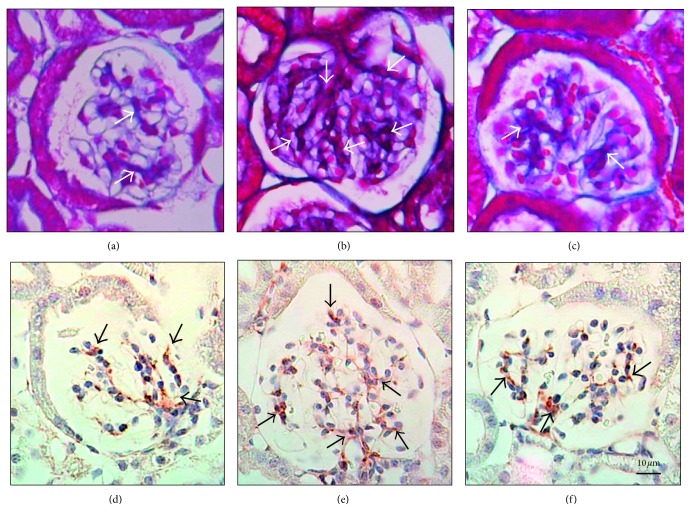
Mesangiolysis in mice. (a)–(c) Azan staining of the glomerulus in the kidney. (a) The glomerulus in the kidney from a normal mouse fed with control diet (CD). Blue regions (white arrows) show collagen fiber. (b) The glomerulus in the kidney from a mouse fed with alcohol diet (AD). Blue regions (white arrows) show collagen fiber, which are increased compared to (a). (c) The glomerulus in the kidney from a mouse fed with AD containing *Lb. paracasei*. Blue regions (white arrows) show collagen fiber, which are reduced compared to (b). (d)–(f) Immunostaining of the glomerulus in the kidney. (d) The glomerulus in the kidney from a normal mouse fed CD. A mesangial cell (black arrow) is stained by desmin. (e) The glomerulus in the kidney from a mouse fed with AD. The number of mesangial cells (black arrows) is higher than in the normal kidney (d). (f) The glomerulus in the kidney from a mouse fed AD containing *Lb. paracasei*. The number of mesangial cells (black arrow) is fewer than that of animals fed AD (e).

There were no differences in kidney tissue between the three groups in H&E staining (data not shown). However, more fibrosis was observed in the glomerulus of the kidney in the AD group than in the other two groups in azan stain (Figures 4(a)–4(c)). Immunostaining results confirmed that the brown areas indicating the existence of mesangial cells were increased in the AD group but was almost nonexistent in the CD and LD groups (Figures 4(d)–4(f)). The average of the number of mesangial cells in the glomerulus of the CD, AD, and LD groups was 3.08 ± 1.02, 4.06 ± 1.07, and 3.39 ± 0.90, respectively. The number of mesangial cells in the AD group was significantly higher than those of the CD and LD groups (*p* < 0.001).

## 4. Discussion

We found that live *Lb. paracasei* NFRI 7415 is beneficial for improving liver damage due to chronic alcohol intake [12]. In the current study, we investigated whether heat-killed *Lb. paracasei* NFRI 7415 influenced kidney and bone in mice fed an ethanol-containing diet with stress. The perirenal fat tissue weights of the AD and LD groups were lower than that of the CD group (*p* < 0.05) (Table 2). It is known that chronic alcohol intake and excessive stress accelerate energy metabolism in the body [21]. The final body weight of the AD group demonstrated frequently observed symptoms such as weight loss compared to be in the LD group. Intake of *Lb. paracasei* NFRI 7415 may have prevented enhanced energy metabolism by alcohol intake and excessive stress.

Fermented dairy products such as yogurt utilizing LAB have been reported to lower serum cholesterol concentrations in animals [22]. We also reported that live *Lb. paracasei* NFRI 7415 reduced the plasma T-cho and hepatic T-cho concentration in rats fed an ethanol-containing diet [12]. In this study, LAB were not found to lower plasma lipids. However, in terms of cholesterol-reducing activity, effects against liver lipids and the plasma GOT level were observed in the LD group receiving heat-killed *Lb. paracasei* NFRI 7415. Plasma GOT and GPT are enzymes recognized as indicators of hepatitis, liver cirrhosis, and cardiac infarction [23]. Levels of these enzymes increase with liver cell damage, as in hepatitis. Thus, these results indicate that heat-killed *Lb. paracasei* NFRI 7415 decreased the plasma GOT level and liver cholesterol concentration caused by chronic alcohol consumption.

As described in our previous paper, the live *Lb. paracasei* NFRI 7415 has the capacity to lower fecal T-cho excretion, and it effectively reduced plasma T-cho concentration [24]. Although no significant difference in fecal T-cho concentrations were observed here, the bile acid level of feces in the LD group increased (Table 4). This increase in the bile acid level appears to have been due to bile acid adsorption by LAB in the intestine [25, 26]. Hence, it suggested that heat-killed *Lb. paracasei* NFRI 7415 had strong bile acid adsorption ability in feces.

Restraint stress load causes osteoporosis, due to an increase in the corticosteroid hormone secreted by the cortex of the adrenal gland, and increases bone resorption [27]. Heavy alcohol consumption has been associated with increased risk of bone fracture [3]. It is known that osteoporotic bones are more likely to fracture, and it is important to determine the bone content in mice fed alcohol-containing diets with restraint stress.

In this experiment, the ash content in the bone of the AD group was lower than those of the other two groups (*p* < 0.05) (Table 4). Although there were no significant differences in right femur weight, or in calcium and phosphorus in bone, each average value of the AD group was lower than those of the CD and LD groups. For example, the right femur weight (dry) of the CD, AD, and LD groups was 57.4 ± 6.23 mg, 53.0 ± 7.85 mg, and 56.1 ± 5.56 mg, respectively. Moreover, the plasma calcium of CD, AD, and LD groups were 3.09 ± 0.58 mg, 4.06 ± 0.62 mg, and 3.54 ± 1.46 mg, respectively. These results indicate that some minerals were eluted from the bone of AD group, promoting bone resorption. On the other hand, the plasma calcium of the LD group tended to be lower, suggesting that bone resorption was inhibited by LAB. It was reported that probiotic yogurt containing strains of *Lactobacillus casei*, *Lactobacillus reuteri*, and *Lactobacillus gasseri* increased apparent calcium absorption and bone mineral content in rats [28]. These LAB strains produce certain prebiotics such as oligosaccharides that help new bone tissue to grow. Further investigation of the effects of live *Lb. paracasei* NFRI 7415 as probiotics is needed.

It was observed that the AD group had lower urine volume than the CD group during the experimental period. Kidney function weakness was therefore suspected in the AD group. Fibrosis of the organization occurs to the organ that the whole body is approximately important except the brain. The organ that caused the fibrosis will eventually malfunction. This means that renal function decreases as kidney fibrosis advances [29]. We therefore performed azan staining to observe fibrosis of the kidney. The mesangium domain in the glomerulus of the kidney consists of mesangium cells and mesangium substrates, including type IV collagen. Collagen fiber is stained blue by azan staining. An increase of the mesangium domain was confirmed, by the increase in blue in the glomerulus of the AD group. We then attempted immunostaining using the desmin antibody, which can specifically stain mesangial cells. Many more mesangium cells, colored brown by immunostaining, were observed in the AD group compared to the CD and LD groups.

There are always mesangial cells in the glomerulus of the kidney. They maintain capillary vessels of the glomerulus of the kidney and work to regulate its ability to filter the blood. Most chronic glomerulonephritis involves mesangial proliferative glomerulonephritis (MesPGN), for which the characteristic is an increase of mesangial cells [30]. The causes of MesPGN development remain to be elucidated. It is thought, however, that immunoreaction is the factor that causes this condition. Mesangium proliferative glomerulonephritis developed in the AD group by alcohol intake with stress.

In conclusion, the present investigation shows that heat-killed *Lb. paracasei* NFRI 7415 inhibits mesangial proliferative glomerulonephritis by alcohol intake with stress. Further studies are needed to investigate in more detail with five dietary treatment groups: a control diet (CD) group, an alcohol diet (AD) group, the CD diet with restraint stress group, the AD with restraint stress group, and the AD with restraint stress containing LAB group. This is needed to clarify the relationship between restraint stress and risk of MesPGN.

## Figures and Tables

**Table 1:** Composition of experimental diets.

Ingredient (g/L)	CD^1^	AD^2^	LD^3^
Casein	41.4	41.4	41.4
L-cystine	0.5	0.5	0.5
DL-methionine	0.3	0.3	0.3
Corn oil	8.5	8.5	8.5
Olive oil	28.4	28.4	28.4
Safflower oil	2.7	2.7	2.7
Vitamin mixture^4^	2.5	2.5	2.5
Mineral mixture^5^	8.75	8.75	8.75
Maltose dextrin mixture	115.2	25.6	25.6
Cellulose	10.0	10.0	10.0
Choline bitartrate	0.53	0.53	0.53
Xanthan gum	3.0	3.0	3.0
Ethanol	—	50.0	50.0
*Lb. paracasei* extract	—	—	(10^7^ CFU/g)

^1^CD, control diet; ^2^AD, alcohol diet; ^3^LD, *Lb. paracasei*-containing alcohol diet; ^4^vitamin mixture (g/kg of mix): retinal, 4.8; cholecalciferol, 0.4; thiamine, 24.0; riboflavin, 0.6; pantothenic acid, 0.6; pyridoxine, 0.7; cobalamin, 0.01; menadione, 0.05; nicotinic acid, 3.0; D-calcium pantothenic acid, 1.6; folic acid, 0.2; biotin, 0.02; para-aminobenzoic acid, 5.0; inositol, 10.0; glucose, 949.02; ^5^mineral mixture (g/kg of mix): CaHPO_4_, 500.0; NaCl, 74.0; K_3_C_6_H_5_O_7_·H_2_O, 220.0; K_2_SO_4_, 52.0; MGO, 24.0; MnSO_4_·5H_2_O, 6.77; FeSO_4_·7H_2_O, 4.95; ZnCO_3_, 1.6; CuCO_3_Cu(OH)_2_H_2_O, 0.3; KlO_3_, 0.01; NaSeO_3_, 0.01; CrK(SO_4_)_2_·12H_2_O, 0.55; NaF, 0.06; sucrose, 115.75.

**Table 2:** Food intake, total energy intake, body weight, and liver, kidney, and perirenal fat tissue weight.

Group	CD^1^	AD^2^	LD^3^
Food intake (g/day)	17.2 ± 0.66^a^	10.9 ± 0.95^b^	11.0 ± 0.91^b^
Total energy intake (Kcal)	2040 ± 780^a^	1382 ± 121^b^	1400 ± 116^b^
Total energy intake (Kcal/day)	75.5 ± 2.89^a^	51.2 ± 4.48^b^	51.9 ± 4.29^b^
Final body weight (g)	40.2 ± 1.39^a^	29.8 ± 2.73^c^	34.5 ± 3.41^b^
Liver weight (g/100 g BW)	4.45 ± 1.54	4.85 ± 1.01	5.05 ± 1.86
Kidney weight (g/100 g BW)	0.59 ± 0.08	0.74 ± 0.08	0.68 ± 0.22
Perirenal fat tissue weight (g/100 g BW)	0.88 ± 0.19^a^	0.33 ± 0.12^b^	0.34 ± 0.21^b^

^1^CD, control diet; ^2^AD, alcohol diet; ^3^LD, *Lb. paracasei*-containing alcohol diet. Values represent mean ±SD, *n* = 8. Values not sharing a common superscript letter are significantly different at *p* < 0.05.

**Table 3:** Plasma lipids and liver lipids.

Group	CD^1^	AD^2^	LD^3^
Plasma lipids			
Triacylglycerol (mg/dL)	143.5 ± 23.8	141.0 ± 42.0	176.3 ± 91.6
Total cholesterol (mg/dL)	167.4 ± 33.8^a^	126.2 ± 14.8^b^	179.2 ± 25.5^a^
HDL-cholesterol (mg/dL)	127.9 ± 18.7^a^	90.9 ± 12.9^b^	120.8 ± 23.8^a^
Liver lipids			
Total fat (mg/g)	75.4 ± 18.4	76.9 ± 25.3	79.0 ± 16.4
Triacylglycerol (mg/g)	27.5 ± 12.4	28.6 ± 12.6	33.3 ± 12.0
Total cholesterol (mg/g)	8.69 ± 3.46^ab^	11.1 ± 3.60^a^	6.37 ± 1.80^b^

^1^CD, control diet; ^2^AD, alcohol diet; ^3^LD, *Lb. paracasei*-containing alcohol diet. Values represent mean ±SD, *n* = 8. Values not sharing a common superscript letter are significantly different at *p* < 0.05.

**Table 4:** Bone content, plasma calcium, and fecal lipids.

Group	CD^1^	AD^2^	LD^3^
Bone contents			
Right femur weight (dry) (mg)	57.4 ± 6.23	53.0 ± 7.85	56.1 ± 5.56
Right femur weight (defatted) (mg)	53.6 ± 6.0	49.3 ± 6.76	52.3 ± 5.30
Ash (%)	53.0 ± 1.44^a^	50.9 ± 2.13^b^	54.0 ± 2.67^a^
Calcium (mg/g)	13.3 ± 0.85	12.7 ± 1.07	13.0 ± 0.77
Phosphorus (mg/g)	15.7 ± 1.23	14.6 ± 1.34	15.4 ± 0.92
Plasma calcium (mg/g)	3.09 ± 0.58	4.06 ± 0.62	3.54 ± 1.46
Fecal lipids			
Total fat (mg/g)	48.2 ± 9.30^a^	29.8 ± 10.9^b^	39.9 ± 9.77^ab^
Bile acid (*μ*g/g)	3.54 ± 0.99^a^	2.38 ± 0.56^b^	3.91 ± 0.74^a^
Total cholesterol (mg/g)	1.87 ± 0.43	1.89 ± 0.56	1.90 ± 0.41

^1^CD, control diet; ^2^AD, alcohol diet; ^3^LD, *Lb. paracasei*-containing alcohol diet. Values represent mean ±SD, *n* = 8. Values not sharing a common superscript letter are significantly different at *p* < 0.05.
